# Effect of Post-Deposition Annealing on the Structural Evolution and Optoelectronic Properties of In_2_O_3_:H Thin Films

**DOI:** 10.3390/nano12193533

**Published:** 2022-10-09

**Authors:** Liangge Xu, Jinye Yang, Kun Li, Lei Yang, Jiaqi Zhu

**Affiliations:** 1 National Key Laboratory of Science and Technology on Advanced Composites in Special Environments, Harbin Institute of Technology, Harbin 150080, China; 2 Center for Analysis and Measurement, Harbin Institute of Technology, Harbin 150001, China; 3 Key Laboratory of Micro-Systems and Micro-Structures Manufacturing, Harbin Institute of Technology, No. 2 Yikuang Street, Nan Gang District, Harbin 150080, China

**Keywords:** In_2_O_3_:H, infrared transparent conductive, thin film, atomic layer deposition

## Abstract

An infrared transparent conductive material is a solution to realize the shielding function of infrared windows against electromagnetic waves, by combining the two characteristics of high transmission and conductivity in infrared wavelengths. Indium-hydroxide-doped (In_2_O_3_:H) thin films were prepared by atomic layer deposition method, which can achieve high IR transmission by reducing the carrier concentration on the basis of ensuring the electrical properties. On this basis, the effect of the post-deposition annealing process on the microstructure evolution and optoelectronic properties of In_2_O_3_:H thin films was investigated in this paper. It is demonstrated that the carrier mobility after annealing is up to 90 cm^2^/(V·s), and the transmittance at the 4 μm is about 70%, meanwhile, the carrier concentration after annealing in air atmosphere is reduced to 10^19^ cm^−3^, with a transmission rate of up to 83% at 4 μm. The simulations visualize the shielding performance of the annealed In_2_O_3_:H thin film against radar electromagnetic waves. It provides a guideline for fabricating lightweight, thin, and multi-functional shielding infrared transparent materials in the key fields of spacecraft and high precision electronics.

## 1. Introduction

In the development of optoelectronic systems, the integration of electromagnetic shielding performance, infrared imaging (mid-infrared band), and other functions into the performance of transparent conductive materials are necessary requirements; that is, maintaining good electrical properties while achieving electromagnetic shielding at the same time, in order to meet the requirements of infrared imaging for mid-infrared transmission performance [1,2]. The carrier mobility of ITO films is only 20–30 cm^2^/(V∙s), and the carrier concentration is as high as 10^21^ cm^−3^, which seriously affects the requirements for infrared transmission performance. Therefore, related research mainly focuses on the visible light band [3,4,5,6].

Macco et al. [7,8,9] demonstrate the preparation of high-quality In_2_O_3_:H as a transparent conductive oxide (TCO) at low temperatures, and the solid phase crystallization of In_2_O_3_:H transparent conductive oxide films prepared by atomic layer deposition. Furthermore, the atomic layer deposition (ALD) process of hydrogen-doped indium oxide (In_2_O_3_:H) using indium cyclopentadienyl (InCp) and both O_2_ and H_2_O as precursors is highly promising for the preparation of transparent conductive oxides. It has a high growth per cycle (>0.1 nm), is viable at temperatures as low as 100 ℃, and produces films with exceptional optoelectronic quality after post-deposition crystallization. Kuang and Macco et al. [10] developed a simple and effective plasma-based surface pre-treatment. A remote inductively coupled O_2_ or Ar plasma is used to modify the surface of a-Si:H, thereby promoting the adsorption of InCp on the surface. Wu and Macco et al. [11] studied the crystal growth and doping with hydrogen dopant in this ALD process. It is shown that an amorphous-to-crystalline phase transition occurs in the low-temperature region (100–150 °C), accompanied by a strong decrease in carrier density and an increase in carrier mobility. At higher deposition temperatures (>200 °C), enhanced crystal nucleation and incorporation of carbon impurities lead to a decrease in grain size and even amorphous phases, respectively, resulting in a significant decrease in carrier mobility. It is also found that the doped hydrogen is mainly from the co-reactants rather than from the InCp precursors.

Recently, crystalline oxide semiconductors were proposed to enhance carrier mobility because the disorder-induced sub-gap states can be suppressed via lattice order [12,13]. Yang et al. [14] reported a field-effect mobility value of 60.7 cm^2^ V^−1^ s^−1^ for a thin-film transistor obtained using polycrystalline In–Ga–O annealed at 700 °C. Yusaku et al. [15,16,17] reported a field-effect mobility value of 50.6 cm^2^ V^−1^ s^−1^ for a TFT obtained using hydrogenated polycrystalline In–Ga–O formed via solid-phase crystallization at 300 °C. Furthermore, they demonstrate the high-performance polycrystalline In_2_O_3_:H TFTs using a low-temperature solid-phase crystallization process. The carrier concentration (*N_e_*) of the solid-phase-crystallization-grown In_2_O_3_:H film decreases significantly to 2.0 × 10^17^ cm^−3^ after annealing at 300 °C in ambient air; this *N_e_* value is two orders of magnitude lower than that of the In_2_O_3_ film without H_2_ introduction.

This study proposes a simple material and a simple process to obtain high-performance In_2_O_3_:H thin films: it is hydrogenated polycrystalline In_2_O_3_:H thin films grown via the low-temperature ALD process. Further heat treatments were used to modulate the evolution of the film structure as well as the type and concentration of defective elements. The proposed method achieves high mid-infrared permeability and high carrier mobility, and has great potential for future transparent, flexible electronics, or infrared optoelectronic system applications in complex electromagnetic environments.

## 2. Materials and Methods

**Scheme 1:**
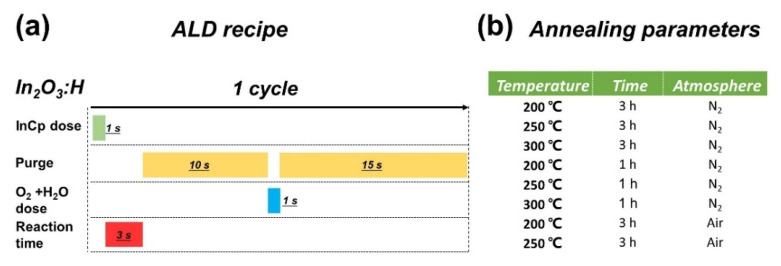
Schematic of the standard recipe (**a**) and annealing process parameters (**b**) for In_2_O_3_:H.

The materials required for the preparation of hydrogen-doped indium oxide films include: InCp metal organic gas source, high-purity Ar (99.99% purity), high-purity oxygen (99.99% purity), high-purity nitrogen (99.99% purity), quartz flakes (10 mm × 10 mm × 1 mm), sapphire flakes (20 mm × 20 mm × 1 mm), deionized water, and anhydrous ethanol (purity 99.7%). InCp was heated to 70 °C and brought into the reaction chamber via Ar gas as a carrier gas. Similarly, to ensure the simultaneous passage of O_2_ and H_2_O, O_2_ was used as the carrier gas and water vapor was brought into the reaction chamber. The prepared In_2_O_3_:H films were post-annealed and the heat treatment process was carried out in nitrogen and air atmosphere (infrared annealing furnace) with treatment temperature intervals of 200–300 °C, both with a temperature gradient of 50 °C and treatment times of one hour and three hours, respectively. The preparation process of H-doped In_2_O_3_ thin films prepared by atomic layer deposition is shown in Scheme 1a. The samples used for annealing were prepared by depositing 600 cycles at 150 °C. These samples were first post-annealed at different annealing temperatures and annealing times under N_2_ protective atmosphere. The specific process parameters are detailed in Scheme 1b.

The crystal structure and phase composition of the films were identified using an X-ray diffractometer (XRD, Empyrean, PANalytical, Malvern, UK), and the average crystal grain size and grain plane spacing were calculated. The thin-film composition was examined by X-ray photoelectron spectroscopy (XPS, ESCALAB 250xi, Thermo Fisher, Waltham, MA, USA). Time-of-flight secondary ion mass spectrometry (TOF-SIMS 5 iontof, PHI NanoTOFII, Okinawa, Japan) was employed to collect depth distribution data of hydrogen elements. The surface structure of the In_2_O_3_ films was studied by atomic force microscopy (AFM, Bruker, Dimension Fast scan, Billerica, MA, USA). Fourier transform infrared spectroscopy (FTIR, PerkinElmer Frontier, Waltham, MA, USA) was used to measure the transmittance of the films in the mid-infrared region, with a measurement range of 2.5 μm to 5 μm and a scan step of 2 nm. The electrical properties were measured using a Hall effect measurement system (HMS-3000, Ecopia, Anyang-city, Korea).

## 3. Results and Discussion

**Figure 1:**
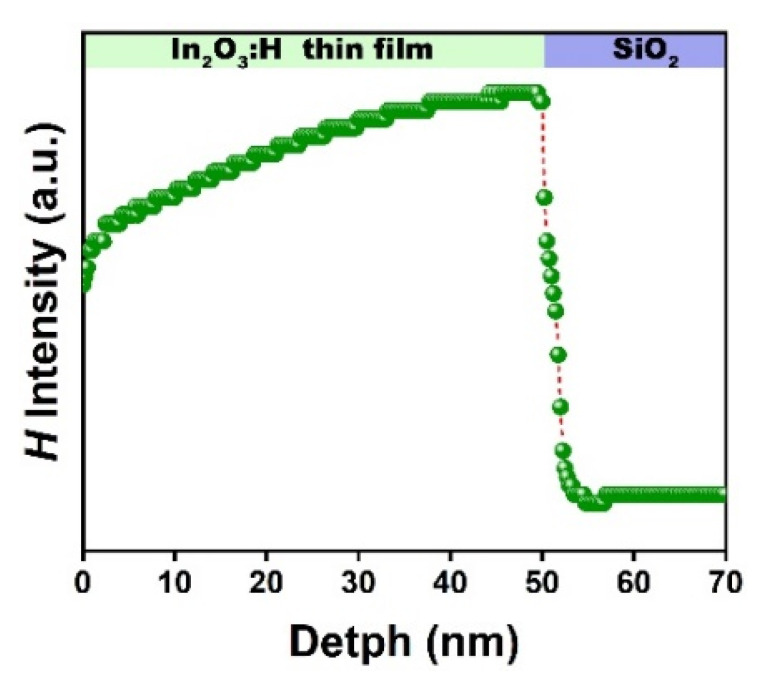
Hydrogen depth profile of the prepared In_2_O_3_:H films.

The SIMS method is the most accurate means of characterizing the amount of hydrogen elements [18,19]. Figure 1 shows the thickness distribution of hydrogen elements in In_2_O_3_:H films prepared by atomic layer deposition, confirming the presence of hydrogen elements. The hydrogen decreases toward the film surface, indicating out-diffusion of the internal hydrogen during the film deposition or after preparation.

### 3.1. Effect of Annealing Temperature and Time on Microstructure

**Figure 2:**
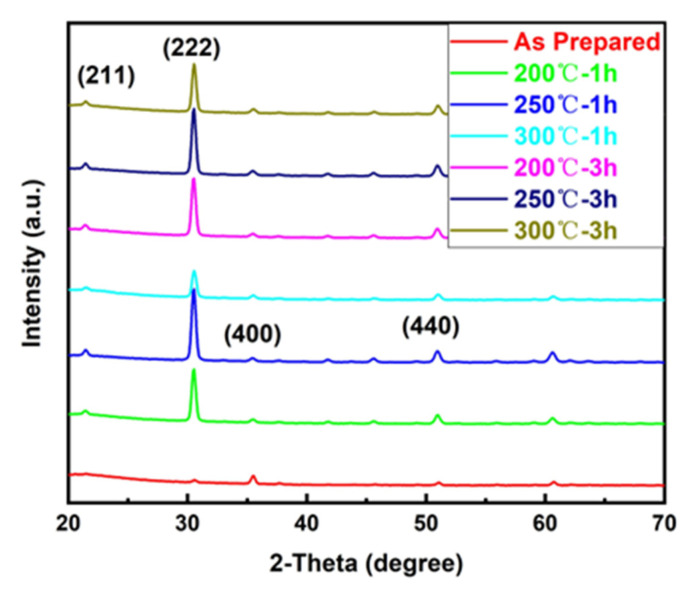
XRD patterns of the prepared In_2_O_3_:H films after annealing at different temperatures and times.

The XRD characterization results of the In_2_O_3_:H films prepared at different annealing temperatures and annealing times are shown in Figure 2. From the figure, it can be seen that the unannealed In_2_O_3_:H films prepared by ALD have some crystalline phase, but the content is still low, and the preferred orientation is mainly in the (400) crystal direction. With the increase in annealing temperature, it can be found that the peak intensity of (222) crystalline direction increases significantly, and the crystallinity increases.

**Figure 3:**
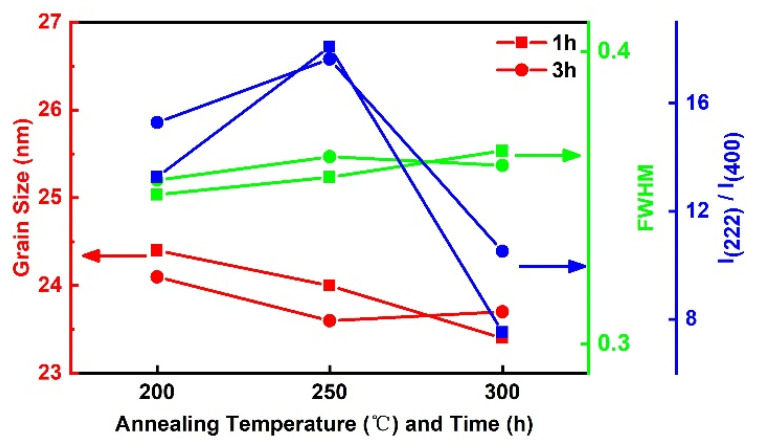
(222)-oriented grain size, diffraction peak half-height width, and relative intensity of (222)/(400) peaks versus annealing temperature and time.

The grain size, half-height width, and relative intensity of the (222)/(400) diffraction peaks for the (222) preferred orientation were further investigated in relation to the annealing temperature and time. As shown in Figure 3, the half-height width of the (222) diffraction peak gradually increases, and the grain size on this orientation gradually decreases as the annealing temperature increases at annealing times of 1 h and 3 h. This may be due to the increase in the Gibbs free energy of the system with the increase in the annealing temperature, which increases the nucleation rate in the film, and finally makes the grain size in the film decrease. In addition, the relationship between the relative intensity of the (222)/(400) peak and the variation of the annealing temperature was analyzed. We found that the relative intensity of the (222)/(400) peaks increases and then decreases as the annealing temperature changes, and the grain content of the (222) orientation is highest at the annealing temperature of 250 °C. From the results in Figure 1, we can find that the preferred orientation of the film before annealing is the (400) orientation, which then changes to the (222) orientation.

**Figure 4:**
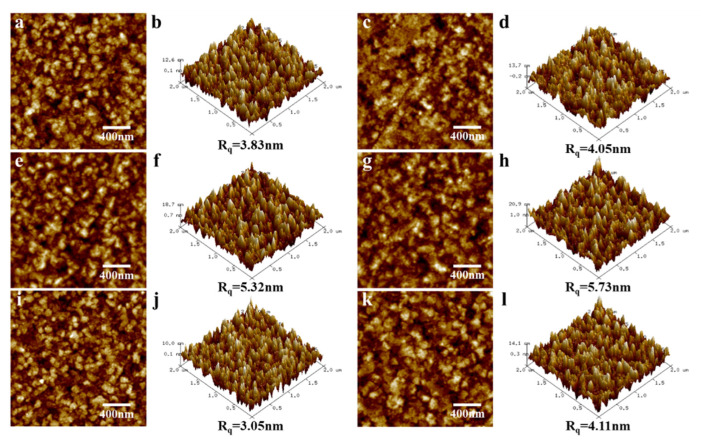
AFM pictures of In_2_O_3_:H thin-film samples at different annealing temperatures and times (**a**,**b**) 200 °C 1 h, (**c**,**d**) 200 °C 3 h, (**e**,**f**) 250 °C 1 h, (**g**,**h**) 250 °C 3 h, (**i**,**j**) 300 °C 1 h, (**k**,**l**) 300 °C 3 h.

Figure 4 shows the AFM characterization results of the In_2_O_3_:H thin-film samples treated at different annealing temperatures and times. It can be seen from the figure that the surface of the annealed sample shows a fine and uniform granular morphology. With the increase in annealing time, the roughness of the film surface slightly increases.

### 3.2. Effect of Annealing Atmosphere on Microstructure

**Figure 5:**
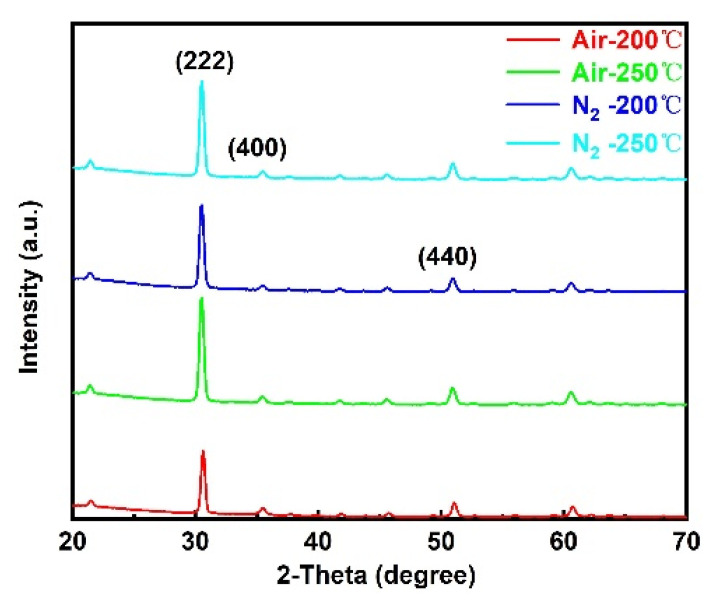
XRD patterns of In_2_O_3_:H films annealed in nitrogen and air atmosphere.

Further, the prepared films were annealed at 200 °C and 250 °C under nitrogen and air atmospheres, respectively, and the XRD test results are shown in Figure 5. It can be seen from the figure that the effect of different annealing atmospheres on the crystal structure is small, the preferred orientation is (222) orientation, and the peak intensity does not change significantly.

**Figure 6:**
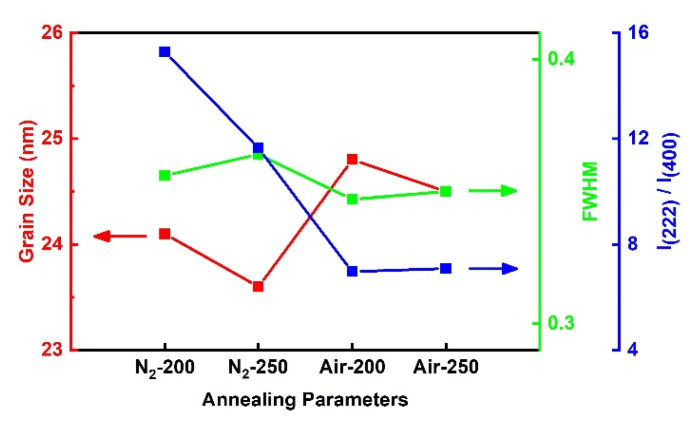
Size of (222)-oriented grains, half-height width of diffraction peaks, and relative intensity of (222)/(400) peaks versus annealing atmosphere.

To further investigate the effect of annealing atmosphere on the microstructure, the grain size, diffraction peak half-height width, and relative intensity of the (222)/(400) peak of the (222) crystal plane were analyzed in relation to the annealing atmosphere, and the results are shown in Figure 6. It can be seen from the figure that the half-height width of the (222)-oriented peak is slightly smaller than that of the sample annealed in N_2_ after the annealing treatment in air, and the grain size of the (222)-oriented is larger than that of the thin-film sample under N_2_ annealing. The relative intensity study of the (222)/(400) peaks show that the grain content of (222)-oriented grains in the films after annealing in the air is slightly lower than that of the films annealed in N_2_. This may be due to the fact that an adequate amount of oxygen is provided during annealing under air, which enters the film during the annealing process and reduces the content of oxygen vacancies. Also, the presence of oxygen may make the (400)-oriented grains smaller in shape and faster in growth rate, and the (400)-orientation in the films after annealing completion is slightly higher than that in the samples annealed in the N_2_ atmosphere.

**Figure 7:**
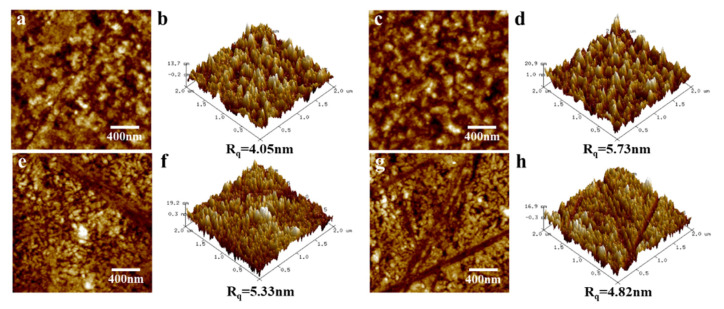
AFM images of In_2_O_3_:H thin−film samples under different annealing atmospheres (**a**,**b**) N_2_ 200 °C, (**c**,**d**) N_2_ 250 °C, (**e**,**f**) air 200 °C, (**g**,**h**) air 250 °C.

Figure 7 shows the AFM characterization results of the In_2_O_3_:H thin film samples under different annealing atmospheres. From the figure, it can be seen that the surface of the films shows fine and uniform granular morphology after annealing in both atmospheres, and the surface roughness does not differ much. However, it can be seen that the grains on the surface of the sample after annealing under air are finer and denser, which we speculate may be because the proportion of (222) orientation in the film after annealing in the air is slightly lower than that of the film sample annealed under N_2_ atmosphere, and the proportion of grains of other orientations on the surface of the sample increases, making the granular morphology of the surface denser.

**Figure 8:**
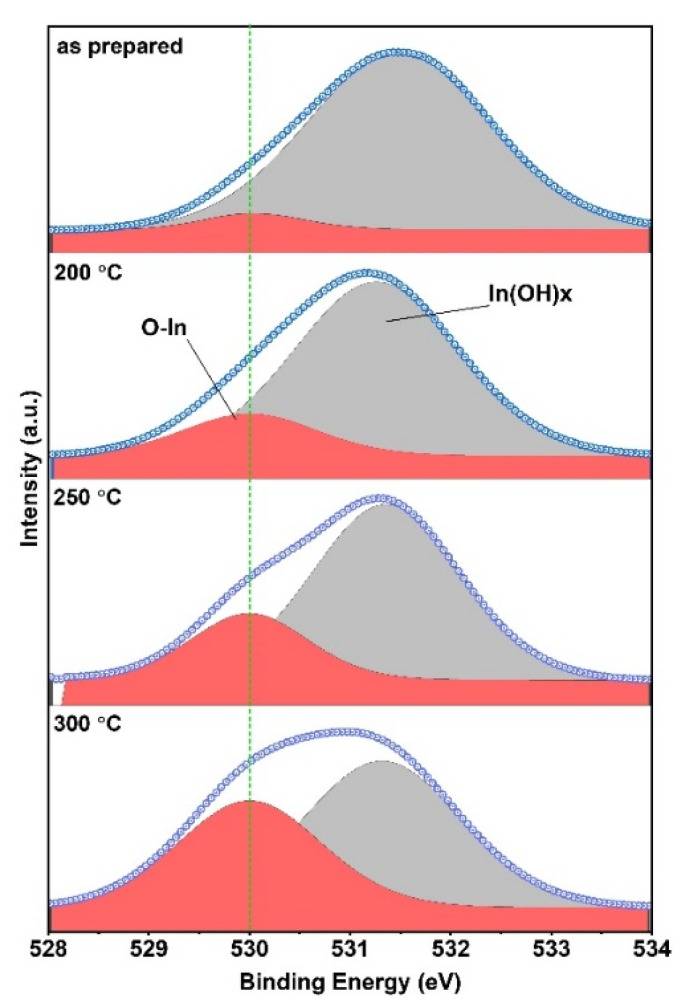
O 1 s XPS core level spectra of the surface-cleaned In_2_O_3_:H films, as prepared and at annealing temperatures of 200, 250, and 300 °C. Al K*α* radiation was used as an excitation source.

XPS analysis of hydrogen in the films can only be used to infer the presence of hydrogen positive ions in the hydroxyl groups [18,20]. Figure 8 represents the O 1 s XPS core level spectra of the In_2_O_3_:H films after surface cleaning, the peak observed at 530 ± 0.1 eV is assigned to the O-In bond and the peak at 531.6 ± 0.3 eV can be attributed to the hydroxyl group, also known as In(OH)_X_, and defects in the film. By increasing the annealing temperature from 200 to 300 °C, the intensity of the O-In peak (Area ratio) increases from 5.66% to 39.76% of the total peak area, while the contribution of the hydroxyl group and the defect state decreases from 94.34% to 60.24%. It is clear that hydrogen positive ions present as hydroxyl groups are the predominant form present in the films grown at low temperatures. Annealing, as well as higher annealing temperatures, favor the reduction in defects, such as hydroxyl groups in the prepared In_2_O_3_ films. Based on the electronic configuration of In, hydroxyl groups, oxygen vacancies, and other possible impurities can lead to shallow donors, which can affect the electrical conductivity.

### 3.3. Effect of Annealing Temperature and Time on Optoelectronic Properties

**Figure 9:**
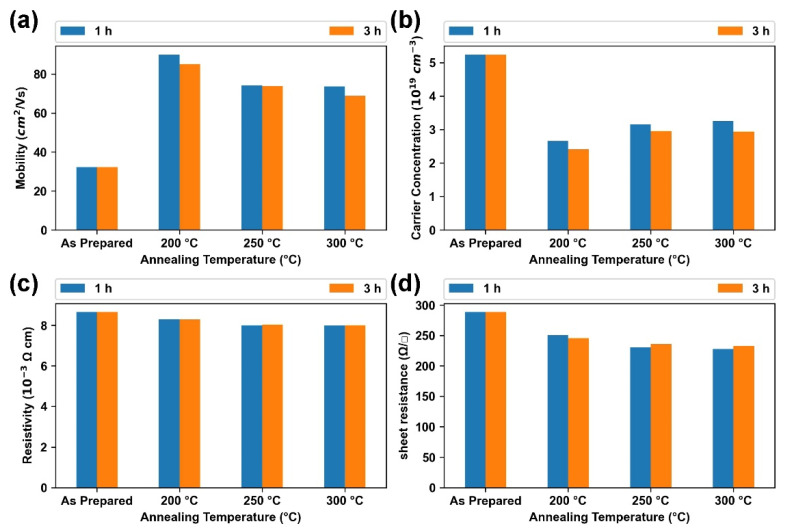
Electrical properties with annealing temperature: carrier mobility (**a**), carrier concentration (**b**), resistivity (**c**), and sheet resistance (**d**).

Figure 9 shows the variation of carrier mobility, carrier concentration, surface resistance, and resistivity with the annealing temperature of the film. With the increase in annealing temperature, the carrier mobility shows a trend of sharp increase and then decrease. As the annealing temperature continues to increase, the carrier mobility starts to decrease because only grain growth occurs in the film at 200 °C annealing, and there is no nucleation effect, i.e., the lower annealing temperature is not sufficient to provide enough Gibbs free energy in the amorphous region of the film to form nuclei. Therefore, annealing at 200 °C yields films with larger grains and fewer internal grain boundary defects, which reduces the migration resistance of free carriers and, thus, increases the carrier mobility. When the annealing temperature is higher than 200 °C, the nucleation effect starts to appear in the amorphous region of the film during the annealing process, and the nucleation starts to grow with time, resulting in smaller grains and more grain boundaries, which increases the free carrier migration resistance and decreases the carrier mobility.

**Figure 10:**
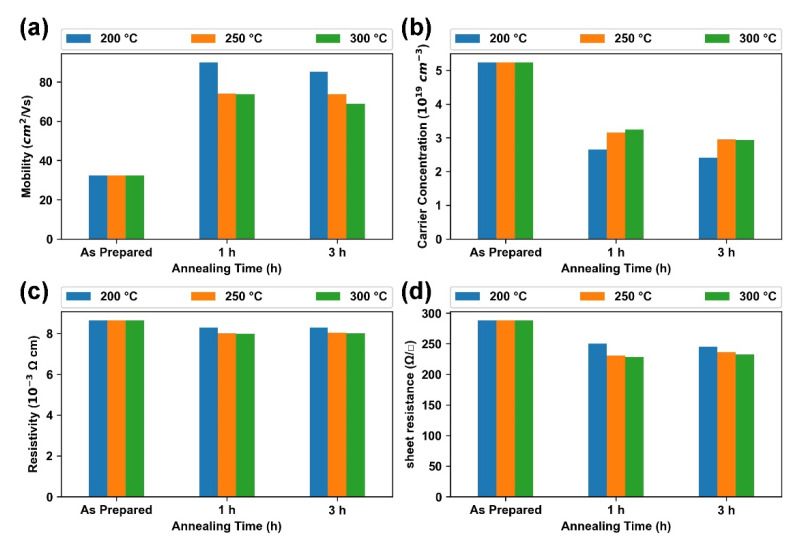
Electrical properties with annealing time: carrier mobility (**a**), carrier concentration (**b**), resistivity (**c**), and sheet resistance (**d**).

Figure 9b shows the variation of carrier concentration with annealing temperature, and it is found that the carrier concentration shows a sharp decrease and then a gradual stabilization with the increase in annealing temperature. Figure 9c,d demonstrate the variation of surface resistance and resistivity with annealing temperature, respectively. Although the carrier mobility increases, the carrier concentration decreases, and the electrical properties become slightly worse, the overall change trend is not significant. Figure 10 shows the variation in electrical properties such as carrier mobility, carrier concentration, surface resistance, and resistivity with annealing time. The mobility decreases with increasing annealing time. This is analyzed in conjunction with the XRD results, which may be caused by grain refinement during the long annealing of the films.

**Figure 11:**
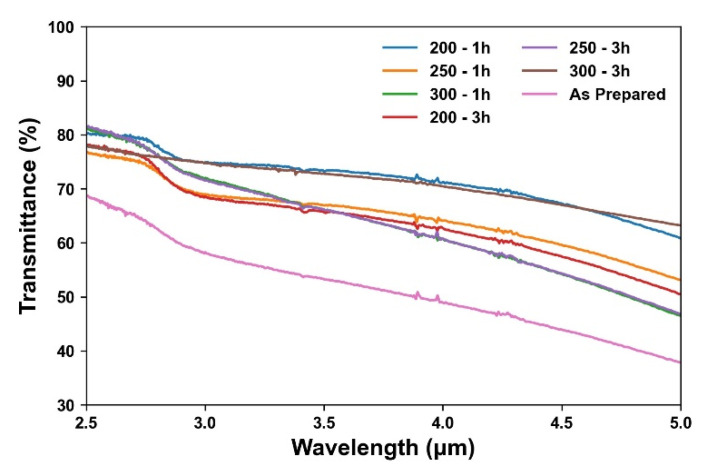
The transmission rate in mid-infrared band: annealing temperature and annealing time.

Figure 11 shows the transmittance of the In_2_O_3_:H films in the mid-infrared band at different annealing temperatures and annealing times. It can be seen from the figure that the transmittance of In_2_O_3_:H films in the mid-infrared band is significantly improved after annealing. This may be due to the decrease in carrier concentration in the In_2_O_3_:H films after annealing, when the scattering of photons by free carriers is weakened and the transmittance increases.

### 3.4. Effect of Annealing Atmosphere on the Photoelectric Properties

**Figure 12:**
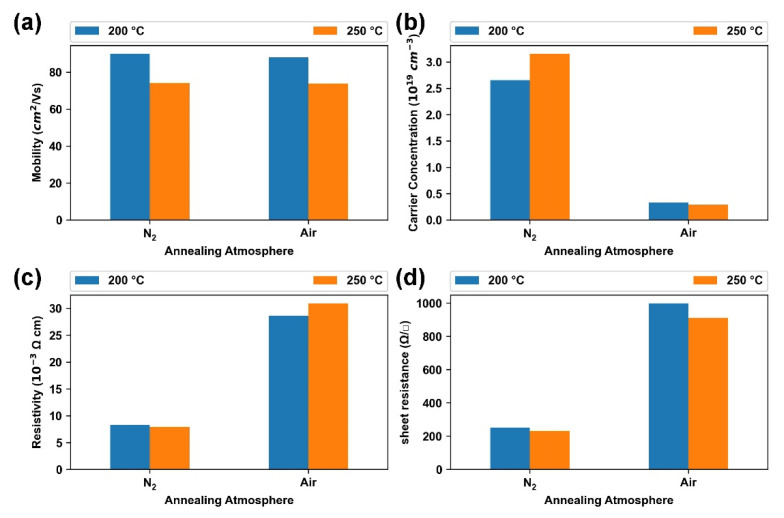
The relationship between electrical properties and annealing atmosphere: carrier mobility (**a**), carrier concentration (**b**), resistivity (**c**), and sheet resistance (**d**).

In order to investigate the effect of annealing atmosphere on the optoelectronic properties, the prepared films were annealed at 200 °C for 1 h and 250 °C for 1 h under the air atmosphere. From the results of Figure 12, it can be seen that the effect of annealing under different atmospheres on the carrier mobility is very small, which can be seen from the structural analysis by XRD, and the crystal structure is basically unchanged before and after annealing at the same temperature atmosphere. In addition, it can be clearly seen that the carrier concentration of the sample decreases sharply from 10^20^ cm^−3^ to 10^19^ cm^−3^ when annealed under air, which is almost an order of magnitude change. This is due to the dramatic decrease in oxygen vacancies inside the sample during annealing in air and the dramatic decrease in carrier concentration, while annealing in the air causes a dramatic decrease in the elemental H content inside the sample due to the reaction with oxygen in the air during annealing.

**Figure 13:**
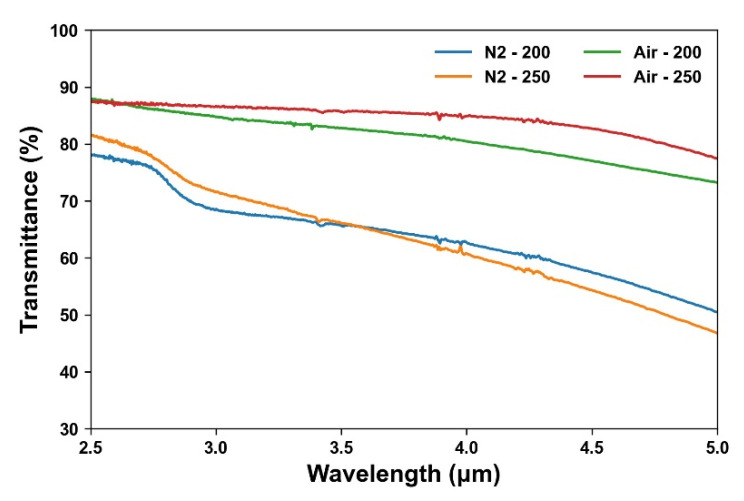
Mid-infrared transmittance of In_2_O_3_:H films in different annealing atmospheres.

Figure 13 shows the trend of the mid-infrared band transmittance of the In_2_O_3_:H films after annealing under two different atmospheres. It can be seen from the figure that the mid-infrared band transmittance of In_2_O_3_:H films after annealing under air increases significantly, with the central wavelength (4 μm) transmittance increasing from about 60% under N_2_ atmosphere to more than 80%. The low carrier concentration attenuates the effect on photons, resulting in an increase in mid-infrared band transmittance.

**Figure 14:**
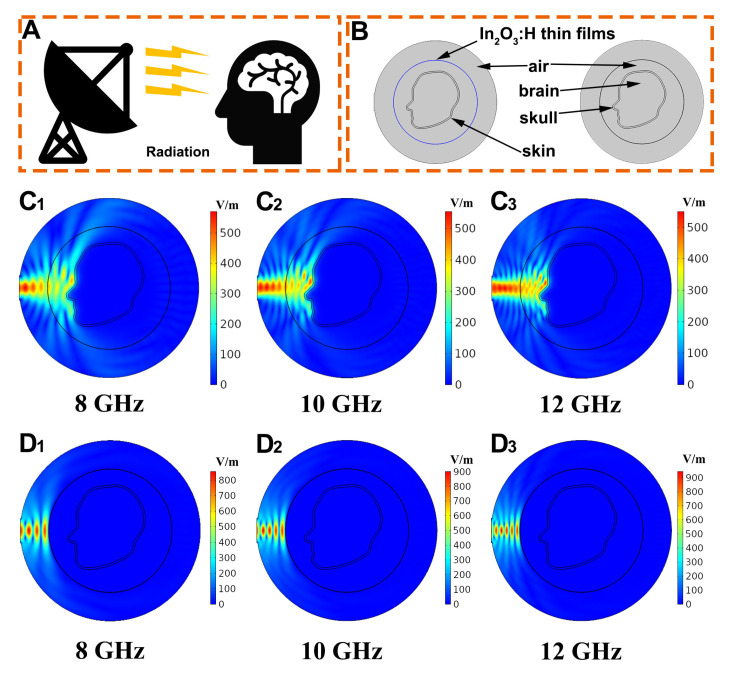
Simulation of the brain exposed to electromagnetic radiation from a radar antenna. (**A**) Schematic diagram of the model; (**B**) simplified radiation model. directly exposed brain and In_2_O_3_:H thin films protected brain; (**C_1_**–**C_3_**) unprotected electric field distribution map; (**D_1_**–**D_3_**) electric field distribution map in In_2_O_3_:H thin-films-protected brain.

To investigate the ability of electromagnetic shielding of In_2_O_3_:H thin films, we used a finite element model to simulate the radiation process of electromagnetic waves generated by the radar antenna to the brain (Figure 14A). The simulation model is shown in Figure 14B. The brain consists of skin, bone, and internal community. The models represent the brain directly exposed to electromagnetic radiation and the brain protected by In_2_O_3_:H films, respectively. The effects of electromagnetic waves at 8 GHz, 10 GHz, and 12 GHz on the brain were selected. It can be seen that the intensity of the radiation electric field is very strong in the air, and the brain protected by In_2_O_3_:H thin films is almost unaffected. The simulation results show that the In_2_O_3_:H thin films we fabricated present a superior ability to shield the external electromagnetic radiation, and effectively achieves electromagnetic shielding.

## 4. Conclusions

Here, we investigated the effect of heat treatment on the microstructural evolution and optoelectronic properties of In_2_O_3_:H films prepared by atomic layer deposition. First, the heat treatment causes a significant increase in the crystallinity of the films, the relative intensity of the (222)/(400) peaks increases significantly, the selective orientation changes from (400) to (222) orientation, and the films annealed at 250 °C yield the highest intensity in the (222) orientation and a slight reduction in grain size with increasing annealing temperature. The crystallinity of the annealed samples becomes better. The carrier mobility of the heat-treated films increases significantly, while the carrier concentration decreases sharply. However, at higher annealing temperatures, the carrier concentration increases slightly; the optimal annealing parameters are annealing at 200 °C for one hour under N_2_ atmosphere. The mid-infrared range’s transmittance reaches more than 80% after one hour at 200 °C. There is no significant change in the mobility of the annealed films under the air atmosphere, but the carrier concentration is reduced by an order of magnitude.
